# MPL S505C enhances driver mutations at W515 in essential thrombocythemia

**DOI:** 10.1038/s41408-021-00583-4

**Published:** 2021-11-29

**Authors:** Leila N. Varghese, Gonzalo Carreño-Tarragona, Gabriel Levy, Xabier Gutiérrez-López de Ocáriz, Inmaculada Rapado, Joaquín Martínez-López, Rosa Ayala, Stefan N. Constantinescu

**Affiliations:** 1 Université Catholique de Louvain and de Duve Institute, Brussels, Belgium; 2 Ludwig Institute for Cancer Research, Brussels, Belgium; 3 WELBIO (Walloon Excellence in Life Sciences and Biotechnology), Brussels, Belgium; 4 Haematology and Haemotherapy Department, Hospital Universitario 12 de Octubre, I+12, CNIO, Complutense University, CIBERONC, Madrid, Spain; 5 Ludwig Institute for Cancer Research, Nuffield Department of Medicine, University of Oxford, Oxford, UK

**Keywords:** Haematological diseases, Haematopoietic cell growth factors, Genomics

**Dear Editor**,

Pathological activation of the thrombopoietin receptor (TPOR/MPL) drives a significant percentage of two of the myeloproliferative neoplasms (MPN), essential thrombocythemia (ET), and myelofibrosis (MF). The single pass helical transmembrane domain of the receptor anchors it to the cell surface and the importance of this domain in controlling the activation of the receptor is evident from both biochemical studies [1, 2] and the identification of several important activating mutations in this region [3–6]. Mutations at the juxtamembrane residue W515 and the transmembrane variant S505N constitute the most common MPL mutations identified in MPN and have been functionally characterized as drivers of pathological activation. However, rarer variants are also found in this region, as well as secondary variants of unknown functional significance.

Here, we report an ET patient negative for JAK2 V617F (qPCR), CALR frameshift mutations (GeneScan), and MPL W515K/L (qPCR). With no family history of MPN, she presented at 83 years of age, after previously having normal platelet levels, and later progressed to post-ET MF. To identify other possible genetic drivers, we performed high sensitivity targeted sequencing of DNA from bone marrow cells as previously described [7]. MPL S505C and W515R mutations were detected *in cis* (Supplementary Fig. 1), verified by Sanger sequencing, with a 0.37 variant allele ratio. Variants after filtering are summarized in Supplementary Table 1. MPL S505C has previously been identified together with W515L in JAK2 V617F-negative ET [8], although the functional consequences of this double mutation have not been established. A recent study by Bridgford and colleagues used a deep sequencing-based saturation mutagenesis approach to identify a number of secondary transmembrane domain mutations at W515—including R and L—that enhance pathological signaling driven by S505N [9]. Likewise, we wanted to test whether the mutation to cysteine at amino acid 505 would similarly enhance the two patient-derived double mutations: W515L, which has been observed *in cis* with S505C in two previous studies and has not yet been functionally characterized, as well as W515R from our patient.

We introduced the S505C, W515L, and W515R mutations individually into the pMX-IRES-GFP HA-MPL WT plasmid, as well as constructing the compound S505C/W515L and S505C/W515R variants, using the Quikchange method (Agilent). As STAT5 is utilized in signaling processes by MPL, we used a Dual Luciferase Reporter assay (Promega) to examine STAT5 transcriptional activity in HEK 293T cells downstream of mutant MPL (Fig. 1B). We found that the S505C did not drive constitutive STAT5 signaling. W515R induced a very small signal in HEK293T cells that were not statistically significant, although we have previously observed it to be weakly active in gamma2A cells. Together, S505C and W515R were able to induce autonomous STAT5 activity. S505C also enhanced W515L-induced autonomous STAT5 activity. In a factor-free proliferation assay using the IL3-dependent Ba/F3 cell line retrovirally transduced with pMX-IRES-GFP HA-MPL plasmids to overexpress MPL WT and mutants, and sorted for equivalent expression of GFP, we found that cells expressing MPL W515R alone became factor independent (Fig. 1C), indicating the small signal in luciferase assay is valid. The MPL S505C variant was not sufficient for factor-independent growth. Only cells expressing W515R/L mutants could proliferate in the absence of cytokine, and that when compounded with the S505C mutation the rate of proliferation increased. As observed in the Bridgford study, W515L was more strongly activating than W515R in cytokine-free conditions [9].

**Fig. 1:**
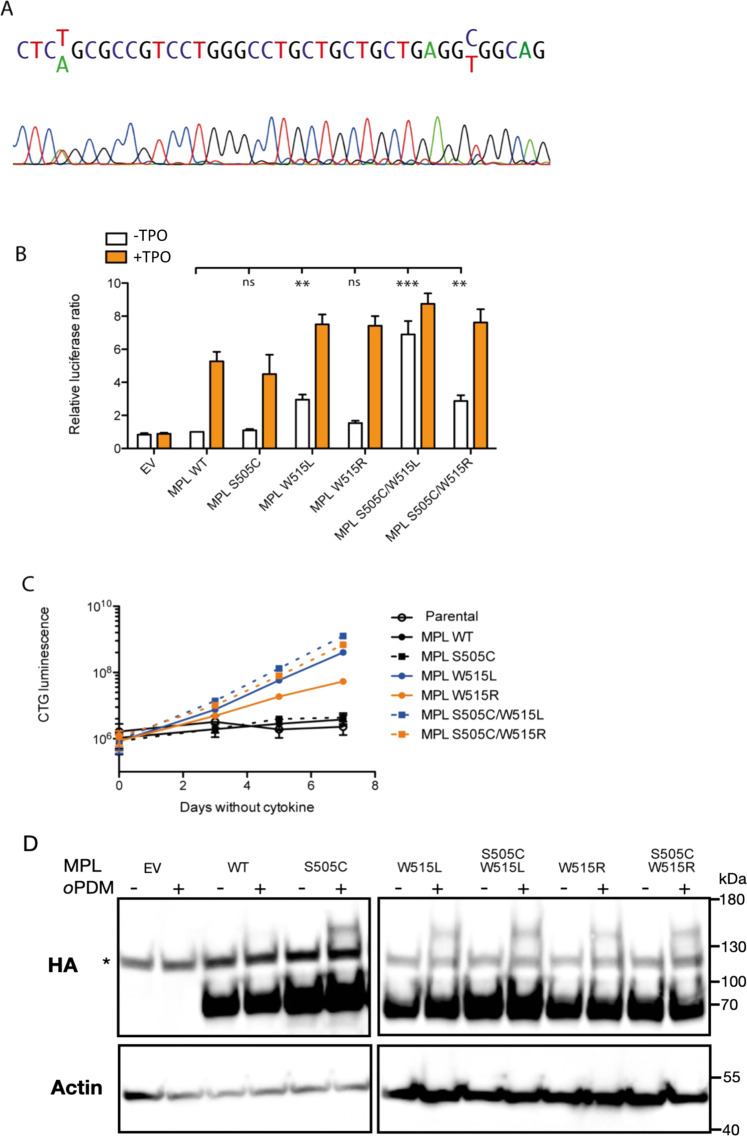
MPL S505C is found together with W515R in ET/MF and enhances autonomous activation of W515R and W515L in vitro. **A** Patient Sanger sequencing demonstrating heterozygous S505C and W515R mutations. **B** Dual luciferase assay for STAT5 transcription activity of MPL variants with and without TPO stimulation. HEK 293T cells were transiently transfected with plasmids for the expression of HA-tagged MPL WT or variants, or pMX-IRES-GFP empty vector (EV), and treated or not with 10 ng/mL recombinant human TPO. Relative luminescence was determined using a Dual Luciferase Reporter Assay system (Promega). Mean ± SD is shown for *n* = 3 independent experiments performed in triplicate, normalized to relative luciferase activity with overexpression of MPL WT in the absence of cytokine stimulation. *: *p* < 0.05, **: *p* < 0.01, ***: *p* < 0.001, by Student’s t-test. **C** Factor-free proliferation assay in Ba/F3 cells. BaF3 cells were transfected with viral supernatants from HEK293T cells to express HA-tagged MPL WT or variants. Stable lines over expressing MPL were selected by flow cytometry-based on cell surface HA expression. Stable cell lines were then cultured for seven days in factor free conditions. Cell viability was determined using the Cell Titer Glo assay (Promega) on days 0, 3, 5, and 7. **D**. Cross-linking of MPL variants in the absence of TPO. A truncated form of MPL, lacking cytoplasmic region (1-560AA) was expressed in CHO cells. 48 h post transfection, cells were treated or not with 100 µM *o*PDM in DMSO for 10 min. Lysates were examined by Western blot for HA-MPL expression with an antibody directed towards HA (C29F4, Cell Signaling). * non-specific band. All cell lines used were routinely tested for mycoplasma contamination.

We have previously shown by solid state NMR of MPL transmembrane and juxtamembrane domains that whereas wildtype human MPL is largely monomeric in the absence of TPO, mutations such as W515L enhance dimerization of these domains. Therefore, to test the effects of S505C alone, and together with W515R/L, on MPL dimerization, we examined whether dimers of HA-tagged MPL S505C could be observed in the absence of stimulation when CHO cells overexpressing truncated MPL were treated with *o*-phenylene dimaleimide (*o*PDM). This crosslinking reagent conjugates cysteines with a spacer arm of approximately 7 Å, maintaining stable dimers under denaturing conditions. This technique allows us to compare levels of dimerization for MPL variants by SDS-PAGE and western blot. A MPL construct with its cytoplasmic domain truncated was used to avoid the confounding effects of crosslinked intracellular cysteines. MPL is mostly monomeric when the wildtype receptor is treated with the crosslinking reagent (Fig. 1D), in line with previous NMR results. However, more stable MPL S505C dimers (approximately 140 kDa MW) were able to form in cells treated with *o*PDM in the absence of TPO stimulation, as we also see with the W515L/R mutants of the receptor, as well as with the double mutants: W515L/S505C and W515R/S505C.

Taken together, our findings suggest that S505C induces receptor dimerization, but that this is not sufficient for signaling to drive autonomous proliferation. Not all dimeric interfaces are conducive to a productive signal, however, the precise relationship between dimeric interface and activation is known only for murine MPL [1, 2], and not the human homolog. Our results suggest MPL S505C alone does not form a dimer in the correct orientation for autonomous signaling. Nonetheless, when compounded with the known pathogenic W515 variants, S505C augments pathological signaling, likely through enhancing MPL dimer formation in a productive conformation for JAK2 activation.

Our study highlights the importance of functional characterization of patient variants of unknown significance, demonstrating that while non-canonical variants such as MPL S505C may not be activating alone—as the more comprehensive Bridgford study corroborates—they might still serve to modify driver mutations, with potentially clinically relevant consequences.

## Supplementary information

Supplemental material
