# Supplementary material for: MPL S505C enhances driver mutations at W515 in essential thrombocythemia
Source: Blood Cancer J. 2021 Nov 29;11(11):188. doi: 10.1038/s41408-021-00583-4 (PMC8630145; doi:10.1038/s41408-021-00583-4)
Supplement: Supplementary file 1 — Supplemental material [file 41408_2021_583_MOESM1_ESM.docx]

**Supplementary materials**

| **Gene** | **Transcript** | **Ref/Variant** | **Allele Coverage** | **Coding** | **Protein** |
| --- | --- | --- | --- | --- | --- |
| MPL | NM_005373.2 | A/T | 5435/3181 | c.1513A>T | p.S505C |
| MPL | NM_005373.2 | T/C | 5509/3167 | c.1543T>C | p.W515R |
| TET2 | NM_001127208.2 | G/C | 570/24 | c.3866G>C | p.C1289S |
| ATRX | NM_000489.4 | CCT/TGG | 361/92 | c.4337_4339delAGGinsCCA | p.E1446_E1447delinsAK |
| PHF6 | NM_032458.2 | T/C | 243/11 | c.158T>C | p.V53A |

Supplementary Table 1.

Variants detected by high sensitivity targeted sequencing.


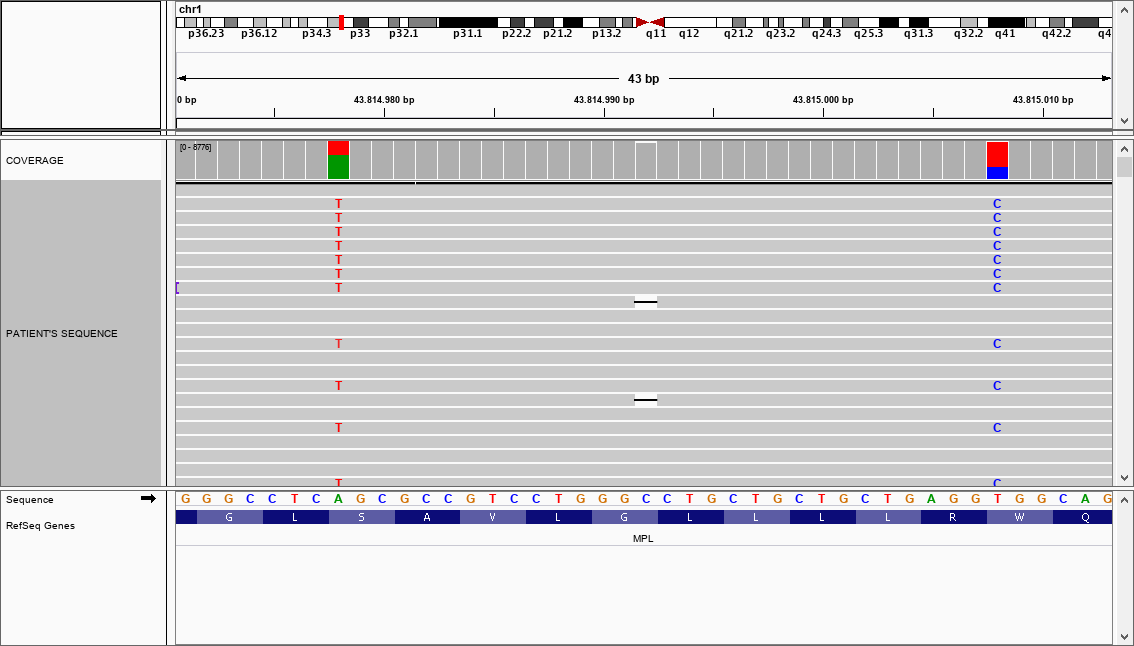


Supplementary Figure 1. Patient’s sequence track showing S505C W515R *cis* mutations
